# The use of Multiple Displacement Amplified DNA as a control for Methylation Specific PCR, Pyrosequencing, Bisulfite Sequencing and Methylation-Sensitive Restriction Enzyme PCR

**DOI:** 10.1186/1471-2199-8-91

**Published:** 2007-10-16

**Authors:** Simon Hughes, J Louise Jones

**Affiliations:** 1 Tumour Biology Laboratory, John Vane Science Centre, Cancer Research UK Clincial Centre, Queen Mary's School of Medicine and Dentistry, UK

## Abstract

**Background:**

Genomic DNA methylation affects approximately 1% of DNA bases in humans, with the most common event being the addition of a methyl group to the cytosine residue present in the CpG (cytosine-guanine) dinucleotide. Methylation is of particular interest because of its role in gene silencing in many pathological conditions. CpG methylation can be measured using a wide range of techniques, including methylation-specific (MS) PCR, pyrosequencing (PSQ), bisulfite sequencing (BS) and methylation-sensitive restriction enzyme (MSRE) PCR. However, although it is possible to utilise these methods to measure CpG methylation, optimisation of the assays can be complicated due to the absence of suitable control DNA samples.

**Results:**

To address this problem, we have developed an approach that employs multiple displacement based whole genome amplification (WGA) with or without *SssI*-methylase treatment to generate CpG methylated and CpG unmethylated DNA, respectively, that come from the same source DNA.

**Conclusion:**

Using these alternately methylated DNA samples, we have been able to develop and optimise reliable MS-PCR, PSQ, BS and MRSE-PCR assays for CpG methylation detection, which would otherwise not have been possible, or at least have been significantly more difficult.

## Background

The major epigenetic alterations in eukaryotes are DNA methylation and histone acetylation. Promoter methylation has an important role in controlling the binding of transcription factors and other proteins to the DNA, which in turn modulate the association of methyl-DNA-binding proteins and histone deacetylases to the transcription start sites. This modulation is critical in regulating the switch between transcriptionally active euchromatin (unmethylated) and transcriptionally silent heterochromatin (methylated) and in turn gene expression [1,2]. The most common methylation event is the addition of a methyl group to the cytosine present in the CpG (cytosine-guanine) dinucleotide [3]. These dinucleotides exist as either CpG islands or as sparsely distributed CpG motifs within the promoter regions of many genes. Hypermethylation (methylation) of these islands or motifs results in transcriptional silencing [4], whilst hypomethylation (demethylation), either global or gene specific, induces expression [5].

PCR-based techniques can be used to investigate the methylation status of CpG islands or motifs with the available methods being categorised based on the requirement for bisulfite treatment prior to PCR (or sequencing). Bisulfite treatment converts all unmethylated cytosine to uracil/thymine, while methylated cytosines are retained. MS-PCR, PSQ or BS can then be used to measure cytosine conversion or retention and thus distinguish methylated from unmethylated residues [6,7]. As an alternative to bisulfite-based approaches, methylation-sensitive restriction endonucleases, which contain one or more CpG motifs within their recognition site, can be employed [8,9]. These enzymes will only cut the DNA if the cytosine within the CpG motif is unmethylated. For this assay, the DNA (non bisulfite treated) is first digested and then subjected to amplification by PCR (MSRE-PCR) using primers flanking the site of interest. If the CpG is methylated, then a PCR product will be generated, however, if there is no methylation, no product will be generated as the site will have been cut.

When designing methylation detection assays using MS-PCR, BS, PSQ or MSRE-PCR optimisation of the amplification conditions, including primer design, magnesium chloride concentration and annealing temperature, is essential to ensure correct interpretation of results. To enable this, suitable control DNA samples are required that correspond to fully CpG unmethylated and fully CpG methylated DNA.

In this paper, we describe an adaptation of the approach described by Weisenberger and colleagues [10]. The methods presented here use a combination of whole genome amplification (WGA) using the multiple displacement amplification (MDA) approach [11] with or without subsequent treatment with the CpG methylating enzyme *SssI*-methylase (*M.SssI*) to generate matched DNA samples differing in only their CpG methylation. The DNA samples generated using this method can be used as CpG methylation control samples for optimising PCR-based assays, as well as internal controls for all of the steps involved in a methylation detection experiment.

## Results and Discussion

Alterations in DNA methylation status can modulate gene expression in the absence of DNA base changes. Although several PCR-based approaches can be implemented to measure methylation, before these can be reliably used to study patient samples it is first essential to optimise assay conditions. Furthermore, as PCR amplification is often the end point measurement in methylation analysis, it is important to have amplification controls as a way of monitoring the whole experimental process, to ensure each step and treatment has worked optimally. While commercially available universally methylated and unmethylated DNA can be used as controls, these have not always been reliable in our assays. As a consequence, we have developed a procedure for generating CpG methylated and CpG unmethylated DNA from the same source DNA using MDA and *M.SssI *treatment.

**Figure 1:**
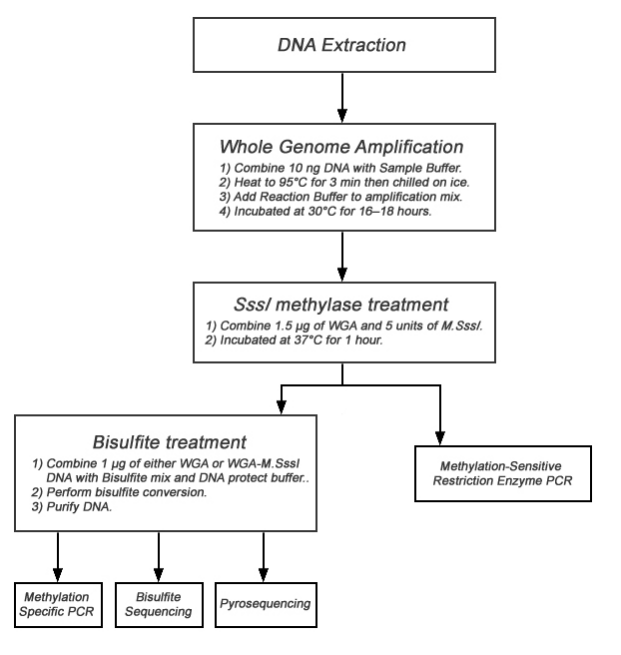
Flow diagram demonstrating the steps involved in generation of differentially methylated DNA and the downstream applications of the DNA.

MDA is a rolling circle amplification method, originally developed for the amplification of large circular DNA templates [12], which has been adapted for the amplification of the entire genome [13,14]. This amplification method can generate DNA strands in excess of 10 kb in length, without prior knowledge of the target template [15]. In the context of this work, MDA generates amplified DNA free of any methylation due to the absence of methylase activity for the MDA enzyme (phi29 polymerase). As a consequence the DNA generated my MDA will be unmethylated DNA (uDNA). *SssI *methylase has been reported to methylate the fifth position of cytosine in all CpG dinucleotides [16], thus the treatment of MDA generated DNA with *M.SssI *will generate CpG methylated DNA (mDNA). A flow diagram of the steps involved is displayed in Figure 1.

**Figure 2:**
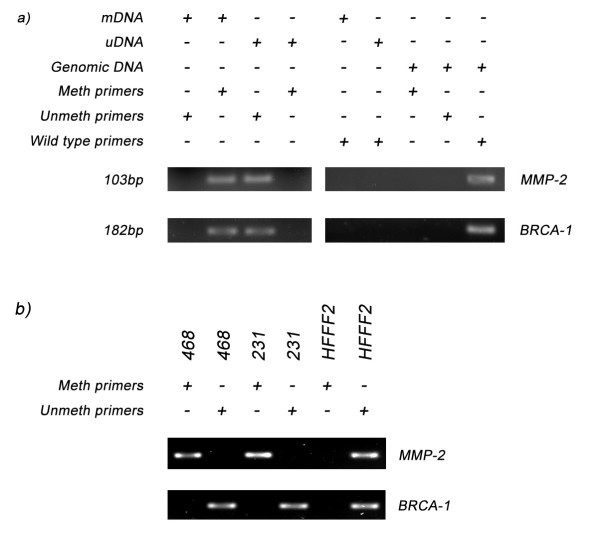
**Methylation Specific PCR results for MMP-2 and BRCA-1**. Two sets of primers were designed for both MMP-2 and BRCA-1, one set that would amplify only methylated DNA and a second set that would amplify only unmethylated DNA. a) Those primers that were designed to amplify methylated DNA only amplified mDNA and not uDNA or genomic DNA, whilst those primers designed to amplify unmethylated DNA only amplified uDNA and not mDNA or genomic DNA. Furthermore wild type primers were unable to amplify either uDNA or mDNA, but could amplify genomic DNA. b) When used in conjunction with cell line DNA they detected that the MMP-2 promoter is methylated for MDA-MB231 (231) and MDA-MB468 (468), but not HFFF2. However, the promoters for MDA-MB231 (231), MDA-MB468 (468) and HFFF2 were all identified as being unmethylated for BRCA-1.

The use of bisulfite treated mDNA and uDNA as template for MS-PCR has allowed for the optimisation of several primer sets. Primers for MS-PCR will ideally only generate a product with either mDNA or uDNA, but not both. Typical results obtained are displayed in Figure 2a. Using MMP-2 and BRCA-1 as examples, the primers that were designed to amplify methylated DNA only amplified mDNA and not uDNA. Conversely those primers that were designed to amplify unmethylated DNA only amplified uDNA and not mDNA. None of the MS-PCR primers sets amplified untreated genomic DNA; in addition wild-type primers did not amplify the bisulfite treated mDNA or uDNA (Figure 2a). When the primers were applied to bisulfite treated DNA from the HFFF2, MDA-MB231 and MDA-MB468 cell lines, all three were demonstrated to be unmethylated for BRCA-1 (Figure 2b). The analysis of MMP-2 methylation status indicated that both MDA-MB231 and MDA-MB468 were methylated, whilst HFFF2 was unmethylated (Figure 2b).

The regions interrogated by MS-PCR for BRCA-1 were also studied by pyrosequencing (PSQ). PSQ, as first described by Ronaghi *et al *[17,18], is a DNA sequencing approach that utilizes a combination of four enzymes (DNA polymerase, ATP sulfurylase, luciferase and apyrase) to perform DNA synthesis in real time (for a review of this technology see [19]). As applied to methylation detection, PSQ can quantify multiple CpG sites per amplicon, whereby the percentage of C bases (methylated) versus T bases (unmethylated) can be calculated for each CpG position in each sample. In order to analyze the BRCA-1 amplicon, studied by MS-PCR, two sets of PCR and sequencing primers (Table 1) were required. The results for uDNA and mDNA demonstrate differential CpG methylation and are in agreement with the MS-PCR results, whereby mDNA is methylated and uDNA is unmethylated. For the eight CpG motifs studied in the uDNA all had undergone 100% bisulfite conversion from C to T, confirming the fully unmethylated status of this DNA as well as indicating that the bisulfite conversion step is working optimally. Similarly, for the eight CpG motifs studied in the mDNA, all of the eight CpG sites showed methylation (C bases retained), with an average of only 25% conversion, indicated by a 75% (non-converted) to 25% (converted) ratio (average over the eight sites) of C to T bases. Thus the *M.SssI *treatment step is 75% efficient at methylating CpG motifs. These findings suggest that although the MDA and *M.SssI *treatment enriches the proportion of methylated DNA, up to 25% of the DNA, within a single sample, may not be methylated at any one of these CpG motifs. Despite this, the results for uDNA and mDNA can be clearly and reproducibly distinguished by PSQ.

**Table 1:** Primer sequences for MS-PCR, PSQ, BS and MSRE-PCR

**Gene**		**Primer Sequence (5' – 3')**	**Technique**	**Methylated (m)/Unmethylated (u)**	**Reference**	**Base pair location**
BRCA-1	F	GGTTAATTTAGAGTTTCGAGAGACG	MS-PCR	m	Genbank: NT_010755.15	5001854 – 5001830
	R	TCAACGAACTCACGCCGCGCAATCG		m		5001697 – 5001673
	F	GGTTAATTTAGAGTTTTGAGAGATG		u		5001854 – 5001830
	R	TCAACAAACTCACACCACACAATCA		u		5001697 – 5001673
MMP-2	F	GGACGTTAAGGGTTTAGAGC	MS-PCR	m	Genbank: NT_010498.15	9127002 – 9127021
	R	CAATACACGACCTCGTCAC		m		9127086 – 9127104
	F	GGATGTTAAGGGTTTAGAGT		u		9127002 – 9127021
	R	CAATACACAACCTCATCAC		u		9127086 – 9127104
BRCA-1-PSQ- PCRa	F	TAGGGGGTAGATTGGGTGGTTA	PSQ		Genbank: NT_010755.15	5001871 – 5001850
	R	CCCCCTCCAAAAAATCTCA				5001675 – 5001656
BRCA-1-PSQ-Sa		TGGGTGGTTAATTTAGAGT				5001859 – 5001841
BRCA-1-PSQ-PCRb	F	TGAGAGTAGGGGTTTAGTTATTTGAGAA	PSQ			5001614 – 5001641
	R	TTTCTATCCCTCCCATCCTCTAATTAT				5001795 – 5001821
BRCA-1-PSQ-Sb		TTTGTTTTTAGTTTAGGAAG				5001651 – 5001670
MMP-14	F	TTGTAATTGGATTTAGGTTAAAA	BS		Genbank: NT_026437.11	4305511 – 4305533
	R	AACACTAAACTTAAATTCCTAAACC				4305741 – 4305765
MMP-1	F	CCAGGCCTCAGTGGAGCTA	MSRE-PCR		Genbank: NT_033899.7	6233232 – 6233214
	R	AATGGGAAGACATTCTCACGA				6233000 – 6232982
MMP-3	F	CAACTTCAAAGCATCTGCTAATT	MSRE-PCR		Genbank: NT_033899.7	6277588 – 6277566
	R	ATGGGCAGAATAGAACAAAGAGG				6277355 – 6277333

**Figure 3:**
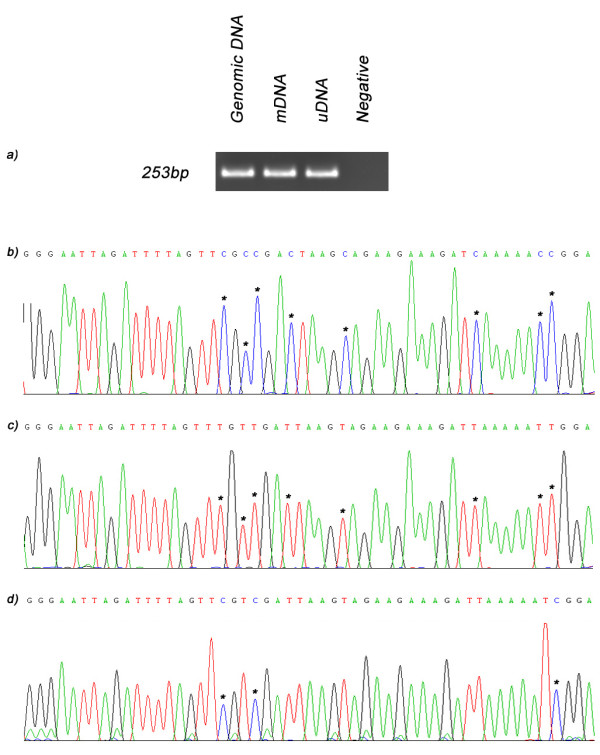
**Bisulfite sequencing results for MMP-14**. a) When genomic DNA (lane 1) and bisulfite treated mDNA (lane 2) and uDNA (lane 3) were used as template for sequencing in combination with primers for MMP-14 a PCR product was generated for all samples but not the negative control (lane 4). Sequencing results for b) non-amplified genomic DNA, c) uDNA and d) mDNA demonstrate that MDA treatment generates DNA (uDNA) free of all methylation as when it is bisulfite treated all cytosine are converted to thymine [indicated by asterix (*)]. In addition, sequencing also demonstrates that *M.SssI *treatment (mDNA) methylates CpG motifs as cytosines are retained when present as part of a CpG dinucleotide (indicated by *).

When genomic DNA and bisulfite-treated mDNA and uDNA were used as template for sequencing in combination with primers for MMP-14, a PCR product was generated for all samples (Figure 3a). BS primers were designed so that they were capable of amplifying the region of interest from all samples, irrespective of methylation status. This was made possible by ensuring that the primers did not contain potential CpG sites that may be prone to methylation. When these PCR products were sequenced, comparison of the results from genomic DNA, mDNA and uDNA allowed discrimination between cytosines that had been methylated (protected from bisulfite conversion and thus remaining as cytosines) or unmethylated (unprotected and converted to thymine) (Figure 3b–d). These bases are indicated in Figure 3b–d by asterix (*). In untreated genomic DNA (Figure 3b) all cytosines are retained, however, in the MDA-generated uDNA (Figure 3c), following bisulfite treatment, all cytosines are converted to thymine. Whilst for the *M.SssI *treated sample, mDNA (Figure 3d), only those cytosines in CpG dinucleotides remain unchanged indicating that they are methylated.

**Figure 4:**
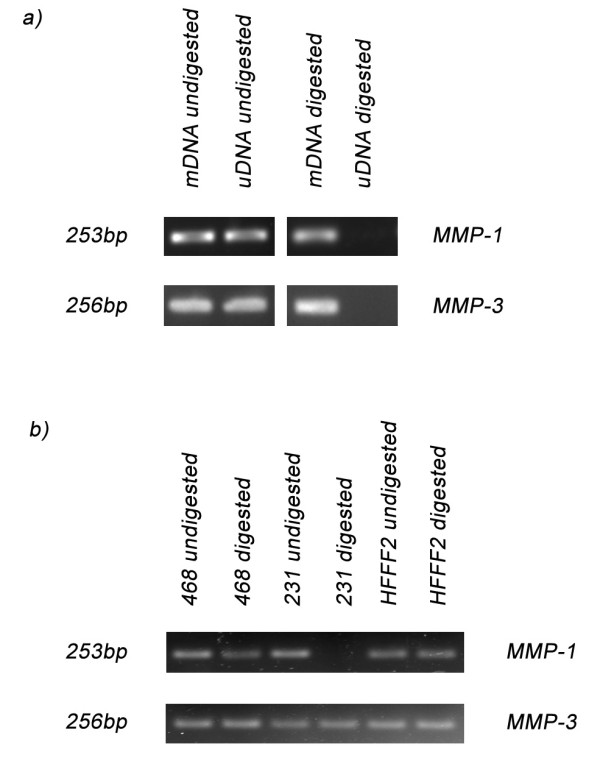
**Methylation-Sensitive Restriction Enzyme PCR for MMP-1 and MMP-3**. a) PCR using primers spanning the restriction site for MMP-1 and MMP-3 gave a PCR product with mDNA but not with uDNA. In contrast, undigested samples gave PCR products for both mDNA and uDNA. b) PCR using digested DNA from MDA-MB231 (231), MDA-MB468 (468) and HFFF2 identified that the CpG motif is methylated for all three cell lines in the MMP-3 amplicon, but only for MDA-MB468 (468) and HFFF2 for the MMP-1 amplicon, with the MDA-MB231 (231) being unmethylated. However, the undigested DNA gave a PCR product with all three cells lines.

The results of the MSRE-PCR using mDNA and uDNA are shown in Figure 4a. The promoter regions of both MMP-1 and MMP-3 have a low proportion of CpG dinucleotides. Associated with some of these motifs are recognition sites for restriction enzymes that are sensitive to CpG methylation (e.g. *HpyCH4IV*, *HpaII*, *SsiI*), whereby when present methylation blocks the enzymes from cutting. Digestion of uDNA with *HpyCH4IV *resulted in cutting of DNA at unmethylated CpG motifs, however, mDNA that possesses methylated CpG motifs remained intact. Subsequent PCR, using primers spanning the restriction site, gave a PCR product with mDNA, indicating CpG methylation and protection, but not with uDNA, showing absence of CpG methylation and sensitivity to digestion. When HFFF2, MDA-MB231 and MDA-MB468 cell line DNAs were subjected to *HpyCH4IV *digestion and MMP-1 and MMP-3 PCR the results demonstrated that the CpG site in the MMP-1 amplicon was methylated in HFFF2 and MDA-MB468, but unmethylated in MDA-MB231 (Figure 4b). The observations for MMP-3 indicated that the CpG site is methylated in all three cell lines (Figure 4b) as all gave a PCR product. Digestion negative samples gave PCR products for all DNA samples (Figure 4a and 4b), proving that the MSRE-PCR results were specific for detection of methylation status and not a failure in the amplification reaction.

## Conclusion

The results demonstrate that the combination of MDA and *M.SssI *treatment generate DNAs that differ only in their CpG methylation status. The examples described herein illustrate uDNA and mDNA can act as methylation-status specific controls for both assay optimisation and as internal controls for methylation experiments. This is important for several reasons; (i) it enables primer and reaction optimisation, (ii) it allows for an experimental checkpoint, for instance ensuring bisulfite conversion is complete, as PCR products will be generated from mDNA and uDNA with both sets of methylation status detection primers if the conversion is incomplete, or for MSRE-PCR to ensure complete digestion and (iii) if mDNA and uDNA controls are processed along side test samples when the controls give expected results then the results obtained from the test samples should be more reliable.

## Methods

### DNA extraction

Genomic DNA was extracted using the Qiagen DNA Mini-kit (Qiagen, Crawley, UK) from normal breast tissue obtained following breast reduction surgery, following ethics approval from the North East London LREC. DNA was also obtained from the cell lines HFFF2, MDA-MB231 and MDA-MB468, using the same technique. DNA concentration was determined using the Nano-drop spectrophotometer (NanoDrop Technologies, Wilmington, USA). Both procedures were performed following manufacturer's instructions.

### Primer design

The process of primer design for MS-PCR and BS is critical when using these techniques and it is highly recommended to use specialised software as standard approaches and programs will not be sufficient. For this study, we used either Methyl Primer Express version 1.0 (Applied Biosystems, Foster City, USA) for primer design (MMP-2 and MMP-14) or utilised primers reported previously [BRCA-1 [20]]. Primer design for pyrosequencing was performed using the PSQ assay design software version 1.0.6 (Biotage, Uppsala, Sweden), whilst primer design for MSRE-PCR can be performed using standard primer design programs. All primers were obtained from Sigma-Aldrich (Gillingham, UK)

### Whole Genome Amplification

DNA was amplified using the GenomiPhi Amplification Kit (Amersham Biosciences, Little Chalfont, UK) according to manufacturer's instructions. Briefly, amplification was carried out in two individual steps. The step 1 reaction mixture contained 5–10 ng of DNA in 1 μl of sterile water and 9 μl of Sample Buffer. This mixture was heated at 95°C for 3 minutes and then chilled on ice. Step 1 results in denaturation of the genomic DNA template. The step 2 reaction (amplification) mixture contained 9 μl of Reaction Buffer, 1 μl of Enzyme Mix and the 10 μl from Step 1. The amplification reaction was incubated at 30°C for 16–18 hours. Step 2 allows for binding of the exonuclease resistant random hexamers and subsequent isothermal amplification. The enzyme was inactivated by heating at 65°C for 10 minutes, followed by cooling to 4°C.

### Assessment of amplification and purification

Five microlitres of each amplification reaction was electrophoresed through a 1% agarose gel and stained with ethidium bromide in order to assess product yield and product length. Amplification products were purified using the QIAquick PCR Purification Kit (Qiagen) and DNA concentration was determined using a Nano-drop spectrophotometer.

### CpG methylation

CpG motifs within the WGA DNA were methylated using the CpG Methylase, *M.SssI *(New England Biolabs, Hitchin, UK) according to manufacturer's instructions. Briefly, 1.5 μg of WGA DNA was combined with 2 μl of 10x NEBuffer 2, 0.1 μl of S-adenosylmethionine (SAM), 5 units of *M.SssI *and sterile water up to a final volume of 20 μl. The reaction was incubated at 37°C for 1 hour, before being purified using the QIAquick PCR Purification Kit and the DNA concentration determined using a Nano-drop spectrophotometer.

### Bisulfite treatment

DNA was bisulfite treated using the EpiTect Bisulfite Kit (Qiagen) according to manufacturer's instructions. Briefly, 1 μg of either CpG methylated WGA DNA, unmethylated WGA DNA or cell line DNA in 20 μl of water was combined with 85 μl of Bisulfite mix and 35 μl of DNA protect buffer. The bisulfite DNA conversion was performed using the following conditions; denaturation 5 min 99°C, incubation 25 min 60°C, denaturation 5 min 99°C, incubation 85 min 60°C, denaturation 5 min 99°C, incubation 175 min 60°C, hold 20°C. The bisulfite converted DNA was purified following manufacturer's instructions. Briefly, the bisulfite reaction was mixed with 560 μl of Buffer BL, applied to the spin column and centrifuged at 12,000 rpm for 1 min. The flow through was discarded and the column washed with 500 μl of Buffer BW. Buffer BD (500 μl) was applied to the column and incubated at room temperature for 15 min. The column was centrifuged to remove Buffer BD and then washed twice with Buffer BW (500 μl). Residual BW buffer was removed by an additional spin (12,000 rpm, 1 min). Buffer EB (20 μl) was added to the column to elute the DNA. The DNA concentration was determined using a Nano-drop spectrophotometer.

### Methylation-specific PCR

PCR was carried out in a 25 μl volume containing 25 ng of either CpG methylated and bisulfite treated WGA DNA (fully methylated), bisulfite treated WGA DNA (fully unmethylated) or cell line DNA, 1 μl of each primer (Table 1) (2 mM stock) for either BRCA-1 (methylated or unmethylated) or MMP-2 (methylated or unmethylated), 2 μl of 2.5 mM dNTP mix (Invitrogen, Carlsbad, USA), 2.5 μl of 10x PCR buffer, 1.25 μl of 50 mM MgCl_2, _0.1 μl of Platinum Taq DNA polymerase (5 U/μl) (Invitrogen) and sterile H_2_O up to a final volume of 25 μl.

Amplification was performed using a "Touchdown PCR" approach, conditions were as follows: initial denaturation for 2 min at 95°C; 20 cycles of denaturing for 30 sec at 94°C, annealing for 30 sec starting at 65°C and decreasing by 0.5°C/cycle and elongation for 30 sec at 72°C; followed by 15 cycles of denaturing for 30 sec at 94°C, annealing for 30 sec at 55°C and elongation for 30 sec at 72°C; then 10 min at 72°C. Five microlitres of each amplification reaction was electrophoresed through a 1% agarose gel and stained with ethidium bromide in order to analyse results.

### Pyrosequencing

PCR was carried out in a 25 μl volume containing 25 ng of either CpG methylated and bisulfite treated WGA DNA (fully methylated) or  bisulfite treated WGA DNA (fully unmethylated) or cell line DNA, 1 μl of each primer (PSQ-PCR; Table 1) (2 mM stock) for BRCA-1, 2 μl of 2.5 mM dNTP mix (Invitrogen), 2.5 μl of 10x PCR buffer, 2.5 μl of 50 mM MgCl_2, _0.1 μl of Platinum Taq DNA polymerase (5 U/μl) (Invitrogen) and sterile H_2_O up to a final volume of 25 μl.

Amplification was performed using the following conditions: initial denaturation for 2 min at 95°C; 45 cycles of denaturing for 30 sec at 94°C, annealing for 30 sec at 58°C and elongation for 30 sec at 72°C; then 10 min at 72°C. Five microlitres of each amplification reaction was electrophoresed through a 1% agarose gel and stained with ethidium bromide in order to analyse results. Using the PCR products as template, PSQ reactions were performed using the BRCA-1 PSQ-S primers (Table 1) and the SQA reagent kit (Biotage, Uppsala, Sweden), following manufacturer's instructions. The results were analyzed using a Biotage PSQ 96MA pyrosequencing system with dedicated Pyro Q-CpG software (Biotage).

### Bisulfite sequencing

PCR was carried out in a 25 μl volume containing 25 ng of either CpG methylated and bisulfite treated WGA DNA (fully methylated), bisulfite treated WGA DNA (fully unmethylated) or non-amplified and untreated genomic DNA, 1 μl of each primer (Table 1) (2 mM stock) for MMP-14, 2 μl of 2.5 mM dNTP mix (Invitrogen), 2.5 μl of 10x PCR buffer, 1.25 μl of 50 mM MgCl_2_, 0.1 μl of Platinum Taq DNA polymerase (5 U/μl) (Invitrogen) and sterile H_2_O up to a final volume of 25 μl.

Amplification was performed as described above with the PCR products being purified using the QIAquick PCR Purification Kit. Using the PCR products as template, cycle sequencing reactions were performed using the MMP-14 Forward and reverse primers and the BigDye Terminator Version 3.1 Kit (Applied Biosystems) following manufacturer's instructions. The results were analyzed using an ABI Prism 3130XL Applied Biosystems DNA sequencer.

### Methylation-Sensitive Restriction Enzyme PCR

CpG methylated WGA DNA, unmethylated WGA DNA or cell line DNA was digested with *HpyCH4IV*, following manufacturer's instructions. PCR was carried out in a 25 μl volume containing 25 ng of digested DNA (or undigested DNA as control), 1 μl of each primer pair (2 mM stock) for either MMP-1 or MMP-3, 2 μl of 2.5 mM dNTP mix (Invitrogen), 2.5 μl of 10x PCR buffer, 1.25 μl of 50 mM MgCl_2_, 0.1 μl of Platinum Taq DNA polymerase (5 U/μl) (Invitrogen) and sterile H_2_O up to a final volume of 25 μl.

Amplification was performed as described above and 5 μl of each amplification reaction was electrophoresed through a 1% agarose gel and stained with ethidium bromide in order to analyse results.

## Authors' contributions

SH designed and carried out the study. SH and JLJ helped prepare the final manuscript for publication. Both authors read and approved the final manuscript.
